# Quality of life in patients with neovascular age-related macular degeneration before and after intravitreal therapy: a longitudinal observational study

**DOI:** 10.3389/fmed.2026.1731481

**Published:** 2026-05-29

**Authors:** Marta Nowak, Anna Maria Cybulska, Aleksandra Derezińska, Elżbieta Grochans, Mariusz Panczyk, Katarzyna Kęcka, Kamila Rachubińska

**Affiliations:** 1 Department of Nursing, Faculty of Health Sciences, Pomeranian Medical University in Szczecin, Szczecin, Poland; 2 Department of Education and Research in Health Sciences, Faculty of Health Sciences, Medical University of Warsaw, Warsaw, Poland; 3 Department of Primary Health Care, Faculty of Health Sciences, Pomeranian Medical University in Szczecin, Szczecin, Poland

**Keywords:** age-related macular degeneration (AMD), anti-vascular endothelial growth factor (anti-VEGF) agents, neovascular age-related macular degeneration (nAMD), quality of life, treatment, vision-related quality of life (VRQoL)

## Abstract

**Background:**

Age-related macular degeneration is a chronic and progressive disease that typically develops after the age of 50. Characterized by visual distortions, image blurring, and gradual central vision loss, age-related macular degeneration significantly impairs patients’ ability to perform everyday activities and contributes to a marked reduction in quality of life. The gold standard treatment for the neovascular form of this condition consists of intravitreal injections. This therapy offers patients the possibility of improved vision-related quality of life by slowing disease progression and partially restoring visual function to levels approximating those prior to the onset of the condition. The aim of this study was to examine vision-related quality of life in individuals with neovascular age-related macular degeneration prior to treatment and following a course of seven intravitreal injections. The study also aimed to analyze its association with visual acuity, contrast sensitivity, and the occurrence and severity of metamorphopsia and central scotoma.

**Methods:**

This survey-based study was carried out using the author’s questionnaire and a standardized research tool—the Polish version of National Eye Institute Visual Functioning Questionnaire. It also involved the analysis of the patients’ medical records.

**Results:**

The study group consisted of 121 patients (121 eyes), comprising 60 women and 61 men, aged between 51 and 90 years. Following a course of intravitreal injections, a statistically significant improvement in vision-related quality of life, as measured by the Visual Functioning Questionnaire, was observed. The analysis also demonstrated an association between quality of life and visual function parameters.

**Conclusion:**

Vision-related quality of life in patients with neovascular age-related macular degeneration was low at baseline and showed a modest improvement following initiation of intravitreal anti-VEGF therapy over the follow-up period. Across time points, better visual acuity, higher contrast sensitivity, and fewer visual disturbances on the Amsler grid were associated with higher vision-related quality of life scores. This study highlights the potential psychosocial benefits of intravitreal therapy in nAMD and may help support patients in initiating treatment or in maintaining adherence to continuing therapy.

## 1 Introduction

Age-related macular degeneration (AMD) is a chronic, progressive retinal disease that primarily affects individuals over the age of 50, with the risk increasing with age (1). Due to rising life expectancy and the widespread adoption of Western dietary and lifestyle habits, the prevalence of AMD continues to increase. It is estimated that by the year 2040, approximately 288 million people worldwide will be affected by the disease. The number of Europeans diagnosed with AMD is expected to reach 21.5 million, of whom 4.8 million will have late stage of the disease, which leads to profound and irreversible visual impairment (2).

Visual disturbances experienced by patients with AMD depend on the stage of the disease. In addition to metamorphopsia—distortion or waviness of the visual image—other symptoms include reduced visual acuity, central scotomas—perception of an oval or irregularly shaped dark spot within the visual field; macropsia—the perception of objects as larger than they actually are; micropsia—the perception of objects as smaller than they actually are; and dyschromatopsia—impaired color perception, which may manifest as colors appearing less saturated than in reality (3).

Quality of life (QoL) is a multidimensional and difficult-to-define concept. An international working group appointed by the World Health Organization (WHO) defines QoL as “an individual’s perception of their position in life in the context of the culture and value systems in which they live and in relation to their goals, expectations, standards, and concerns”. A principle of modern medicine is a holistic approach to patient care—striving for progress not only in the somatic domain but also in psychosocial and spiritual aspects. When two treatment methods yield equivalent clinical outcomes, the preferred approach is the one that improves the patient’s QoL (4).

Due to the undeniable impact of the disease on perceived QoL, the concept of health-related quality of life (HRQoL) was introduced. Health-related quality of life reflects the patient’s subjective experiences related to the effects of the disease and its treatment on functioning in physical, psychological, social, and spiritual domains. It provides insight into the patient’s difficulties not only from a medical perspective (clinical symptoms of the condition) but also from a non-medical perspective (functioning within society). A high HRQoL score indicates good functioning across the assessed life domains, whereas a low score reflects the limitations experienced as a result of the disease (5).

Age-related macular degeneration has a substantial negative impact on patients’ quality of life. The progression of the disease gradually leads to the loss of central vision, impacting the performance of daily activities and psychosocial functioning. Patients report not only a decline in visual acuity but, more importantly, the consequences of this impairment. Everyday tasks become challenging, including grocery shopping, meal preparation, and driving; handling administrative matters; leisure activities such as watching television and reading books; as well as social aspects like recognizing close relatives and interpreting their emotions during conversations (6, 7).

Previous studies have identified that the QoL of patients with AMD is comparable to that of individuals requiring regular kidney dialysis and those who have suffered a massive stroke. Patients struggling with AMD have also reported that they would be willing to give up several years of their remaining life in exchange for improved vision, highlighting the profound impact this condition has on their daily functioning (8, 9).

Currently, no intervention effectively and entirely addresses both the disease and its underlying mechanism. The standard treatment for neovascular age-related macular degeneration (nAMD) is intravitreal therapy with anti-vascular endothelial growth factor (anti-VEGF) agents. This treatment involves intravitreal administration of drugs that reduce intraocular VEGF levels—a factor with pro-angiogenic activity that plays a key role in the development of pathological neovascularization in nAMD. Intravitreal therapy can stabilize or improve vision in a substantial proportion of patients, thereby enhancing the visual prognosis and reducing the incidence of severe vision impairment associated with this condition (10, 11).

While numerous studies have demonstrated that anti-VEGF therapy improves visual outcomes and quality of life in patients with neovascular AMD, several gaps remain in the literature (12, 13). In particular, there is limited real-world longitudinal evidence from Central and Eastern European populations, where healthcare systems, treatment accessibility, and patient characteristics may differ from those in Western countries.

Moreover, relatively few studies have simultaneously examined objective visual function parameters and patient-reported outcomes within a unified analytical framework.

Therefore, the present study aims not only to assess changes in vision-related quality of life following anti-VEGF therapy but also to explore the relationship between VRQoL and key visual function parameters in a real-world clinical setting.

The aim of this study was to assess the vision-related quality of life (VRQoL) among patients diagnosed with nAMD before treatment and after receiving seven intravitreal injections, and to determine how it was related to the values of visual acuity and contrast sensitivity, as well as the presence and pattern of Amsler-grid abnormalities.

## 2 Materials and methods

### 2.1 Study setting and sampling

**FIGURE 1:**
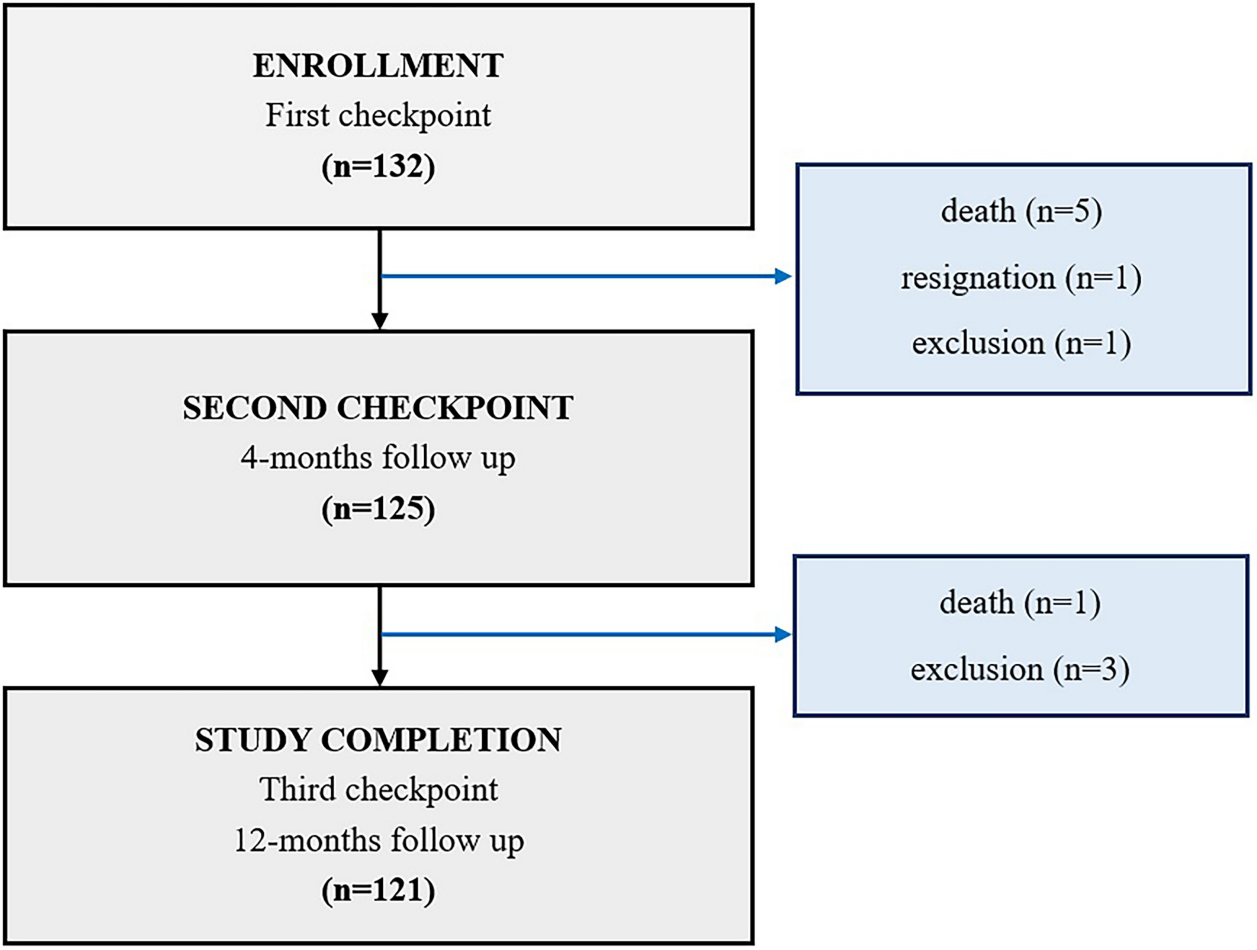
Participant flow diagram of the study.

A longitudinal observational study was conducted. The study was conducted at the University Clinical Hospital No. 2 of the Pomeranian Medical University in Szczecin among patients diagnosed with AMD (ICD code H35.3), who were qualified for treatment under the drug program reimbursed by the National Health Fund. Due to the real-world clinical setting and ethical considerations related to withholding treatment in patients with active neovascular AMD, a control group was not included in the study design. A total of 132 individuals were enrolled; however, six patients died, one withdrew from treatment, and four were excluded from the drug program due to not meeting the therapy criteria. Ultimately, data from 121 patients (121 eyes) were included in the analysis (Figure 1).

The inclusion criteria for the study were: diagnosed nAMD, informed written consent for participation, and agreement to undergo treatment within the drug program titled “Treatment of patients with retinal diseases.” The inclusion criteria for the program were: active subretinal neovascularization affecting over 50% of the lesion, confirmed by optical coherence tomography (OCT) combined with OCT angiography or fluorescein angiography, age above 45 years, total lesion size under 12 disc areas, best-corrected visual acuity between 0.2 and 0.8 in the treated eye (assessed using Snellen or ETDRS charts), informed written consent, and absence of predominant geographic atrophy, hemorrhage, or irreversible foveal damage prior to therapy.

The exclusion criteria for the study were as follows: lack of consent to participate in the research, refusal to undergo treatment within the drug program, psychiatric disorders potentially causing depressive or anxiety symptoms unrelated to AMD, and neurodegenerative diseases (such as Alzheimer’s disease or dementia) that make it impossible for the patient to complete the questionnaire on their own, hypersensitivity to the study medication, active ocular or periocular infection, active endophthalmitis, pregnancy or lactation, occurrence of side effects contraindicating further use of the drug, rhegmatogenous retinal detachment or macular hole of grade 3 or 4, and disease progression defined as a decline in visual acuity below 0.2 lasting more than 2 months or irreversible foveal damage.

### 2.2 Instruments

This survey-based study was carried out using the author’s questionnaire and a standardized research tool, the Polish version of the National Eye Institute Visual Functioning Questionnaire (NEI-VFQ-25). The study additionally made use of the medical records of the participants.

1. The author’s questionnaire consisted of questions related to sociodemographic data (sex, age, education level, marital status, place of residence, and employment status), as well as comorbidities, substance use (alcohol, nicotine), history of ophthalmic and laser procedures, ocular conditions (cataract, glaucoma), and treatment history concerning intravitreal injections.
2. The Visual Function Questionnaire (VFQ-25) used in this study is the Polish adaptation of the National Eye Institute Visual Function Questionnaire (NEI VFQ-25). The questionnaire consists of 25 items grouped into 12 subscales: general health, general vision, ocular pain, distance vision, near vision, social functioning, mental health, role difficulties, dependency, driving, color vision, and peripheral vision. Responses are rated on a five- or six-point Likert scale and scored according to the original scoring key. The VRQoL score is calculated by averaging the total score of all applicable responses, divided by the number of completed items. The higher the score, the better the VRQoL. Minimum clinically important difference (MCID) values for NEI VFQ-25 are 4 points (14).

To facilitate participation among individuals with visual impairment, the questionnaire was formatted using a large font and high-contrast layout. For those who were unable to read the form independently, the questions were read aloud by a researcher, and their responses were recorded accordingly.

In the course of the study, the participants also underwent the following diagnostic evaluations:

1. Distance visual acuity testing from a distance of 5 meters under artificial lighting conditions, performed both without correction and with spectacle correction based on refractive error measurements obtained using an autorefractokeratometer. Illuminated Snellen chart optotypes were used, and the resulting values were converted to logMAR equivalents.

2. Assessment of the presence and severity of metamorphopsia and central scotoma using the Amsler grid (Type 1 chart), conducted at a distance of 30 cm from the face under artificial lighting, with spectacle correction based on refractive measurements obtained via autorefractokeratometry.

3. Contrast sensitivity testing using the Pelli–Robson chart at a distance of 1 meter under artificial lighting conditions, with spectacle correction based on refractive measurements obtained via autorefractokeratometry.

### 2.3 Data collection

Data collection was integrated within a project financed by the National Health Fund and involved two assessment checkpoints. The first assessment (checkpoint) was conducted during the qualification visit preceding the first intravitreal injection. The second assessment was performed at the follow-up visit after the completion of seven intravitreal injections. The first three injections were administered at 28-day intervals, while the remaining four were given at 56-day intervals. At each checkpoint, the patients underwent comprehensive ophthalmic evaluation, including the assessment of visual acuity, contrast sensitivity, OCT and OCT angiography imaging, slit-lamp examination, and completing questionnaires evaluating VRQoL.

All injections were administered at the AMD Clinic, First Department of Ophthalmology, Clinical Hospital No. 2, Pomeranian Medical University in Szczecin, following the treatment protocol established under the 2022–2023 drug program. Each patient received a total of seven intravitreal injections as part of the treatment protocol. The initial loading phase consisted of three injections administered every 28 days, followed by four maintenance injections given at 56-day intervals. The participants were treated with one of two anti-VEGF agents: Aflibercept (2 mg), administered to 111 patients, or Brolucizumab (6 mg), administered to 10 patients. Treatment switching between agents was not allowed; each patient remained on the same drug throughout the course of the study. The choice of anti-VEGF agent was determined by the clinical protocol of the drug program and physician decision, reflecting real-world clinical practice rather than random allocation.

An important methodological consideration in this study is treatment heterogeneity. Patients received different anti-VEGF agents, primarily aflibercept and, to a much lesser extent, brolucizumab. These agents differ in their pharmacological properties, efficacy profiles, and safety considerations. However, due to the substantial imbalance between treatment groups, with the majority of patients receiving aflibercept, subgroup analyses were not performed, as they would lack sufficient statistical power and could lead to unreliable conclusions. Therefore, the findings of this study should be interpreted as reflecting the overall effect of anti-VEGF therapy rather than the comparative effectiveness of specific agents.

### 2.4 Sample size

No formal a priori sample size calculation was performed because this was a longitudinal observational study based on a consecutive sample of all eligible patients treated at the study site during the recruitment period. Therefore, the final sample size was determined pragmatically by the number of patients who met the eligibility criteria and completed both study assessments. A total of 132 patients were enrolled, of whom 121 provided complete data and were included in the final analyses. To improve transparency, an a posteriori sensitivity analysis indicated that, with 121 participants, a two-sided α of 0.05, and 80% power, the study was able to detect a within-subject effect size of approximately *d*_*z*_ = 0.26 for paired comparisons and an overall multiple-regression effect size of approximately *f*^2^ = 0.09 for models including three predictors.

### 2.5 Data analysis

Statistical analyses were performed using Statistica 13.3 (TIBCO Software Inc., Palo Alto, CA, United States). Descriptive statistics included means, standard deviations, medians, and ranges. Because the study comprised two assessment points only (baseline before the first intravitreal injection and follow-up after completion of seven injections), within-patient changes in vision-related quality of life were assessed using paired-samples *t*-tests. These analyses were performed for the NEI VFQ-25 total score and for each VFQ-25 subscale, and 95% confidence intervals for mean differences were calculated. To account for multiplicity across the paired comparisons for the VFQ-25 total score and 12 subscales, Bonferroni correction was applied, with an adjusted significance threshold of *p* < 0.0038 (0.05/13).

To examine associations between visual function parameters and vision-related quality of life, two separate multiple linear regression models were fitted: one at baseline and one after completion of seven intravitreal injections. In both models, the NEI VFQ-25 total score was treated as the dependent variable, whereas uncorrected visual acuity, corrected visual acuity (logMAR), and contrast sensitivity were entered as independent variables. Prior to model estimation, the assumptions of linear regression were evaluated, including normality of residuals, homoscedasticity, and absence of problematic multicollinearity. Regression results are reported as unstandardized regression coefficients (b), standard errors (SE), standardized coefficients (β), 95% confidence intervals, t statistics, the overall model F statistic, and adjusted R^2^.

To compare mean NEI VFQ-25 scores across Amsler-grid categories (no visual disturbances, metamorphopsia, scotoma, and both scotoma and metamorphopsia), one-way analysis of variance was conducted separately at baseline and follow-up. When the assumption of homogeneity of variances was not met, Welch’s ANOVA was used. Significant omnibus tests were followed by Games–Howell *post hoc* pairwise comparisons. Effect sizes for group comparisons are presented as partial eta-squared (η^2^).

Analyses were conducted on complete cases, i.e., participants with data available at both study checkpoints. A two-sided *p*-value of < 0.05 was considered statistically significant.

### 2.6 Ethical consideration

The study was conducted in accordance with the principles of the Declaration of Helsinki and was approved by the Bioethics Committee of the Pomeranian Medical University in Szczecin (KB-006/02/2022/Z). Each participant provided written informed consent to take part in the research.

## 3 Results

### 3.1 Characteristics of the sample

A total of 132 individuals were enrolled; however, six patients died, one withdrew from treatment, and four were excluded from the drug program for not meeting the therapy criteria. Ultimately, data from 121 patients (121 eyes) were included in the analysis, including 60 women and 61 men, aged between 51 and 90 years. Both the mean age and the median age of the participants were 75 years. The largest age groups (each accounting for 23.97% of the sample) were those aged 66–70 and 71–75 years, followed closely by participants aged 76–80 years (23.14%). The majority of the participants were in legally recognized relationships (63.64%), while 36.36% were single. More than half of the respondents (58.67%) lived in cities with a population of over 100,000; nearly one-third (29.75%) lived in cities with 10,000 to 100,000 inhabitants. The smallest groups were residents of towns with fewer than 10,000 inhabitants (5.79%) and rural areas (5.79%). Most participants were retired (86.78%), and a small proportion (10.74%) reported being employed while retired. A total of 121 eyes were examined—65 right eyes (46.28%) and 56 left eyes (53.72%). Regarding smoking status, 18 participants (14.88%) reported being active smokers, while 103 (85.12%) denied tobacco use. The most prevalent comorbidities were hypertension (32.23%) and a combination of diabetes and hypertension (26.45%). Only 17 respondents (14.05%) reported having no diagnosed chronic medical conditions.

### 3.2 VRQoL as measured by the NEI VFQ-25

The analysis of vision-related quality of life using the NEI VFQ-25 questionnaire showed that, prior to treatment, the mean score obtained by the participants was 50.72 ± 27.09. After receiving seven intravitreal injections, this score increased to 61.10 ± 26.61, with a maximum possible score of 100. This change exceeds established thresholds for clinical relevance (4 points). Both before the initiation of treatment and following the seven anti-VEGF injections, the lowest scores were recorded in the subscales of mental health (37.40 ± 30.29 vs. 49.85 ± 29.16), general health (39.05 ± 22.10 vs. 40.50 ± 20.98), general vision (40.83 ± 19.39 vs. 54.42 ± 18.94), distance vision (43.09 ± 20.65 vs. 54.34 ± 20.81), near vision (44.10 ± 22.74 vs. 55.35 ± 23.25), and role difficulties (48.76 ± 24.18 vs. 60.23 ± 22.11).

The highest scores were noted in the subscales of color vision (83.25 ± 22.49 vs. 88.50 ± 20.85), ocular pain (75.93 ± 17.84 vs. 82.54 ± 16.81), social functioning (65.48 ± 22.63 vs. 73.16 ± 22.10), peripheral vision (57.64 ± 21.37 vs. 67.98 ± 19.42), driving (53.57 ± 28.83 vs. 64.86 ± 27.87), and dependency (52.34 ± 31.72 vs. 64.60 ± 29.27) (Table 1).

**TABLE 1:** Changes in vision-related quality of life (VRQoL) across NEI VFQ-25 subscales before and after intravitreal therapy.

VFQ-25 subscales		*M*	SD	Δ	95% CI		*t*	*p* *
					lower	upper		
General health	1	39.05	22.10	−1.45	−3.79	0.90	−1.221	0.224
	2	40.50	20.98					
General vision	1	40.83	19.39	−13.59	−16.60	−10.59	−8.956	**< 0.001**
	2	54.42	18.94					
Ocular pain	1	75.93	17.84	−6.61	−8.38	−4.84	−7.371	**< 0.001**
	2	82.54	16.81					
Near vision	1	44.10	22.74	−11.25	−13.03	−9.47	−12.441	**< 0.001**
	2	55.35	23.25					
Distance vision	1	43.09	20.65	−11.26	−12.94	−9.57	−13.134	**< 0.001**
	2	54.35	20.81					
Social functioning	1	65.48	22.63	−7.68	−9.72	−5.64	−7.419	**< 0.001**
	2	73.16	22.10					
Mental health	1	37.40	30.29	−12.45	−14.39	−10.51	−12.602	**< 0.001**
	2	49.85	29.16					
Role difficulties	1	48.76	24.18	−11.47	−13.47	−9.47	−11.300	**< 0.001**
	2	60.23	22.11					
Dependency	1	52.34	31.72	−12.26	−14.30	−10.21	−11.806	**< 0.001**
	2	64.60	29.27					
Driving	1	53.57	28.83	−11.29	−14.16	−8.41	−7.749	**< 0.001**
	2	64.86	27.87					
Color vision	1	83.25	22.49	−5.25	−7.72	−2.78	−4.214	**< 0.001**
	2	88.50	20.85					
Peripheral vision	1	57.64	21.37	−10.34	−13.27	−7.39	−6.949	**< 0.001**
	2	67.98	19.42					
Total	1	50.72	27.09	−10.38	−11.02	−9.73	−31.531	**< 0.001**
	2	61.10	26.61					

1, before treatment; 2, after treatment; M, mean; SD, standard deviation; Δ, difference of means; CI, confidence interval; t, paired samples *t*-test; p, statistical significance (*p*-values according to paired-samples t-test; significance for the VFQ-25 total score and subscale comparisons was additionally evaluated using Bonferroni-adjusted α = 0.0038); *– according to paired samples *t*-test. Bold values indicate statistically significant results (two-sided *p* < 0.05).

Statistically significant differences (*p* < 0.001) were observed between the mean VFQ-25 scores before treatment and after the series of seven intravitreal injections across all subscales, except for the General Health subscale. After Bonferroni correction for 13 VFQ-25 outcomes, all previously significant pre–post differences remained statistically significant, whereas the General Health subscale remained non-significant. In all cases, mean scores increased after completion of treatment (Δ < 0), indicating improved quality of life among the patients. The greatest improvements were observed in the subscales of general vision (Δ = −13.59), mental health (Δ = −12.45), dependency (Δ = −12.26), role difficulties (Δ = −11.47), driving (Δ = −11.29), distance vision (Δ = −11.26), and near vision (Δ = −11.25). For clarity, the most clinically relevant changes across VFQ-25 subscales are highlighted in the text, while detailed results are presented in Table 1.

### 3.3 VRQoL according to the NEI VFQ-25 depending on visual acuity and contrast sensitivity

The data analysis demonstrated a statistically significant impact of visual acuity (both uncorrected and corrected) and contrast sensitivity on VRQoL in the study group (*p* < 0.001). These three predictors collectively accounted for 25.9% of the variance in the VFQ-25 scores [*F*(3, 117) = 15.0; *p* < 0.001] prior to treatment initiation. Before treatment, higher corrected visual acuity and higher contrast sensitivity were significantly associated with better VFQ-25 scores: corrected visual acuity (β = −0.27, 95% CI −0.46 to −0.08, *p* = 0.007), uncorrected visual acuity (β = −0.19, 95% CI −0.38 to −0.01, *p* = 0.043), and contrast sensitivity (β = 0.22, 95% CI 0.06 to 0.39, *p* = 0.010) (Table 2).

**TABLE 2:** Associations between visual acuity, contrast sensitivity, and VRQoL, as measured by the NEI VFQ-25 before treatment and after seven intravitreal injections.

Regression model test—before treatment							
Adjusted *R*^2^	*F*		df1	df2		*p*	
0.259	15.000		3	117		**<0.001**	
**Predictor**	***b***	**SE**	**β**	**95%CI lower**	**95%CI upper**	***t***	***p***
Intercept	63.00	9.69	N/A	N/A	N/A	6.500	**< 0.001**
Uncorrected visual acuity	−13.60	6.66	−0.19	−0.38	-0.01	−2.050	0.043
Corrected visual acuity	−26.80	9.76	−0.27	−0.46	-0.08	−2.750	0.007
Contrast sensitivity	16.80	6.38	0.22	0.06	0.39	2.630	0.010
**Regression model test—after receiving seven intravitreal injections**							
**Adjusted *R*^2^**	***F***		**df1**	**df2**		***p***	
0.286	17.1		3	117		** < 0.001**	
**Predictor**	***b***	**SE**	**β**	**95%CI lower**	**95%CI upper**	***t***	***p***
Intercept	53.11	9.89	N/A	N/A	N/A	5.368	< 0.001
Uncorrected visual acuity	−4.86	9.16	−0.06	-0.29	0.17	−0.530	0.597
Corrected visual acuity	−20.48	8.57	−0.29	-0.54	−0.05	−2.390	0.018
Contrast sensitivity	16.09	5.54	0.28	0.09	0.47	2.902	0.004

b, unstandardized regression coefficient; SE, standard error; β, standardized regression coefficient; 95% CI, 95% confidence interval for standardized β; t, test statistic for an individual regression coefficient; F, overall model F statistic; df1/df2, numerator and denominator degrees of freedom; adjusted R^2^, adjusted coefficient of determination. Bold values indicate statistically significant results (two-sided *p* < 0.05).

After the participants received a series of seven intravitreal injections, a statistically significant association of corrected visual acuity and contrast sensitivity on VRQoL was observed (*p* < 0.001). The three analyzed predictors together accounted for 28.6% of the variance in the VRQoL scores according to the VFQ-25 [*F*(3, 117) = 17.1; *p* < 0.001] after receiving seven intravitreal injections. After seven intravitreal injections, better corrected visual acuity (β = −0.29, 95% CI −0.54 to −0.05, *p* = 0.018) and higher contrast sensitivity (β = 0.28, 95% CI 0.09–0.47, *p* = 0.004) remained significantly associated with better VFQ-25 scores, whereas uncorrected visual acuity was not significantly associated with VFQ-25 (β = −0.06, 95% CI −0.29 to 0.17, *p* = 0.597) (Table 2).

### 3.4 VRQoL according to the NEI VFQ-25 depending on the presence and severity of metamorphopsia and central scotoma (the Amsler grid)

The data analysis showed that both before treatment initiation (*p* < 0.001) and after receiving seven intravitreal injections (*p* = 0.012), the patients with visual disturbances on the Amsler grid had a statistically significantly lower mean VRQoL compared to the patients without visual disturbances (Table 3).

**TABLE 3:** Associations between the presence and severity of metamorphopsia and central scotoma (Amsler grid) and VRQoL, as measured by the NEI VFQ-25 before treatment and after seven intravitreal injections.

The amsler grid		VFQ-25		*F*	*p* *	η ^2^
		M	SD			
1	No visual disturbances	61.99	23.12	5.040	< 0.001	0.115
	Presence of metamorphopsia	53.23	18.36			
	Presence of scotoma	42.75	16.63			
	Presence of both scotoma and metamorphopsia	44.66	14.05			
2	No visual disturbances	65.38	18.15	3.830	0.012	0.089
	Presence of metamorphopsia	59.00	17.52			
	Presence of scotoma	47.87	10.94			
	Presence of both scotoma and metamorphopsia	46.82	12.53			

VFQ-25, Visual Functioning Questionnaire-25; 1, measurement before treatment initiation; 2, measurement after receiving seven intravitreal injections; M, mean; SD, standard deviation; p, statistical significance; *, one-way ANOVA with Welch’s correction for heteroscedasticity, η^2^, partial eta-squared. Bold values indicate statistically significant results (two-sided *p* < 0.05).

The patients reporting central scotoma (Δ = 19.24) or both central scotoma and metamorphopsia (Δ = 17.33) showed significantly lower VRQoL scores on the VFQ-25 before starting treatment, compared to those without any symptoms on the Amsler test. After receiving seven anti-VEGF intravitreal injections, the patients experiencing both central scotoma and metamorphopsia still demonstrated a significantly lower QoL (Δ = 18.56) than the patients without any Amsler test abnormalities. No statistically significant differences were observed between the patients reporting only metamorphopsia or only central scotoma and those without any visual disturbances on the Amsler test (Table 4).

**TABLE 4:** Comparison of the Amsler grid test results before treatment and after receiving seven anti-VEGF injections, depending on the absence of visual disturbances, and the presence and severity of metamorphopsia and central scotoma.

Amsler grid test—comparison		Δ	SE	*t*	*p* *
1	No visual disturbances—presence of metamorphopsia	8.76	5.17	1.694	0.557
	No visual disturbances—presence of scotoma	19.24	6.03	3.191	0.011
	No visual disturbances—presence of both scotoma and metamorphopsia	17.33	5.47	3.168	0.012
	Presence of metamorphopsia—presence of scotoma	10.48	4.64	2.256	0.156
	Presence of metamorphopsia—presence of both scotoma and metamorphopsia	8.57	3.89	2.201	0.178
	Presence of scotoma—presence of both scotoma and metamorphopsia	−1.91	4.97	−0.384	1.000
2	No visual disturbances—presence of metamorphopsia	6.38	3.34	1.913	0.228
	No visual disturbances—presence of scotoma	17.51	8.15	2.150	0.144
	No visual disturbances—presence of both scotoma and metamorphopsia	18.56	7.00	2.649	0.045
	Presence of metamorphopsia—presence of scotoma	11.13	8.11	1.372	0.519
	Presence of metamorphopsia—presence of both scotoma and metamorphopsia	12.18	6.97	1.748	0.304
	Presence of scotoma—presence of both scotoma and metamorphopsia	1.04	10.19	0.102	1.000

1, measurement before treatment initiation; 2, measurement after receiving seven intravitreal injections; Δ–difference of means; SE, standard error; p, statistical significance; *, *post-hoc* test for differences between means with Games–Howell’s correction for multiple comparisons.

## 4 Discussion

The present study contributes to the existing literature by providing real-world longitudinal data on vision-related quality of life in patients with neovascular AMD from a Central and Eastern European population. Although the beneficial effects of anti-VEGF therapy have been widely documented, our findings extend current knowledge by integrating clinical and patient-reported outcomes and examining their interrelationships within a standardized treatment regimen (12, 13).

Patients with neovascular AMD experience visual disturbances that affect multiple aspects of daily functioning. Timely diagnosis and prompt initiation of anti-VEGF therapy under the supervision of a professional treatment team offer patients with nAMD the prospect of reducing disease activity and stabilizing visual parameters, ultimately leading to an improvement in their QoL.

The VRQoL of patients diagnosed with nAMD, as assessed by the NEI VFQ-25 questionnaire, was low prior to the initiation of therapy. Similar VRQoL outcomes based on the NEI VFQ-25 were reported by other authors who examined patients with nAMD, subdividing them into three groups according to the disease severity. The lowest scores were observed in patients with bilateral late-stage AMD, followed by those with unilateral late-stage AMD, while the highest scores were noted in patients with early-stage disease. Furthermore, the mean composite VRQoL score was significantly lower across all nAMD subgroups compared to the control group without ocular diseases (15).

In addition to statistical significance, the clinical relevance of the observed improvement in vision-related quality of life was assessed using the minimum clinically important difference for the NEI VFQ-25. This indicates that the improvement is not only statistically significant but also clinically meaningful, reflecting a perceptible enhancement in patients’ visual functioning, better performance in everyday activities and greater overall wellbeing.

These findings support the relevance of the applied treatment approach, highlighting its potential to provide tangible benefits from the patient’s perspective beyond numerical changes in questionnaire scores (12, 13).

The low VRQoL scores obtained in our study were also consistent with findings reported by other researchers, who demonstrated that, in addition to reduced QoL, patients with nAMD showed more than twice (in a Canadian population) and more than ten times (in a German population) greater need for assistance with daily activities, as well as nearly threefold higher incidence of falls, compared to individuals without AMD (16, 17).

Higher VRQoL levels among patients with nAMD were reported by authors studying Scandinavian populations (18, 19). In Norwegian patients newly diagnosed with nAMD, with no prior history of intravitreal therapy, VRQoL was significantly higher than that observed in the present study and was consistent with the QoL levels noted in Danish patients with nAMD. The high and comparable outcomes in Scandinavian populations may be explained by geographic and systemic similarities between the two countries, particularly in terms of their healthcare systems, which are well-equipped to detect the disease at an early stage—before the onset of serious visual consequences.

Patients with nAMD exhibit significantly poorer functioning—particularly in the NEI VFQ-25 subscales related to mental health, general vision, near vision, and distance vision—compared to individuals without ocular disease (20). The decline in QoL of patients with nAMD is proportional to the severity of the disease (21). QoL in mild nAMD is comparable to that of patients with moderate angina or symptomatic HIV infection. In moderate nAMD, QoL resembles that of individuals who have experienced a moderate stroke and require substantial assistance with daily activities. In severe nAMD, QoL is similar to that of patients with end-stage renal disease undergoing home dialysis, whereas in very severe nAMD, QoL approximates that observed after a severe stroke resulting in incontinence and the need for constant care (9, 22).

Neovascular AMD has a significantly greater impact on VRQoL compared to other serious ocular diseases such as diabetic retinopathy, cataract, glaucoma, retinal detachment, or keratoconus. These differences may stem from the fact that in nAMD, the pathology affects the macular region, which is responsible for the highest visual acuity. As a result, in addition to reduced visual acuity and contrast sensitivity—common to the other conditions mentioned—patients with nAMD also experience distressing metamorphopsia and a loss of central vision (14, 23).

The deterioration of visual function leads to difficulties in performing daily activities, contributing to a loss of personal independence. Central vision is essential for tasks such as reading, shopping, cooking, eating, driving, recognizing faces, and completing administrative duties. Although peripheral vision is often preserved in patients with nAMD, it is insufficient to allow for efficient and independent performance of basic daily activities, which in turn results in a diminished QoL (24).

The results of the present study showed that VRQoL, as assessed using the NEI VFQ-25, improved following treatment with seven intravitreal anti-VEGF injections. However, the overall score remained low. A significant improvement in VRQoL—consistent with the findings of the present study—was observed both in the short term (3–6 months) and in the long term (12–24 months) after the initiation of treatment, as confirmed by two comprehensive literature reviews (12, 13).

Different results were reported by researchers analyzing VRQoL among patients with nAMD during a 3-year follow-up. Although improvements in visual acuity and QoL were observed 3 months after treatment initiation, a decline in both visual acuity and QoL was noted after 37 months in the majority of treated patients. These differences may be attributed to a smaller sample size compared to the present study and, as the authors emphasize, to an insufficient number of injections due to the treatment regimen applied (25, 26). For comparison, in the present study, all patients received seven intravitreal injections over a 12-month period.

In the present study, the greatest improvements in VRQoL were observed in three vision-related subscales: general vision, near vision, and distance vision. Significant improvements were also noted in the subscales of mental health, dependency, role difficulties, and driving. These findings are consistent with those of other researchers and confirm that the improvement in VRQoL among patients with nAMD is not limited to visual aspects but also extends to mental wellbeing and social functioning (27, 28).

The results of the present study demonstrated that, both before the initiation of treatment and after receiving a series of seven intravitreal injections, patients with higher visual acuity and better contrast sensitivity reported higher VRQoL. Perceived visual disturbances also played a significant role—patients who exhibited no symptoms on the Amsler grid test reported notably better outcomes than those experiencing a central scotoma, metamorphopsia, or both visual impairments.

The findings of the present study are consistent with results from large real-world studies and registry-based analyses, which have demonstrated that anti-VEGF therapy leads to improvements in both visual function and patient-reported outcomes. However, these studies also emphasize variability in long-term outcomes, often influenced by treatment adherence, follow-up frequency, and healthcare system factors.

In this context, our results support the growing body of real-world evidence indicating that improvements in vision-related quality of life are closely linked to functional visual outcomes, particularly when treatment is delivered in a structured and timely manner.

This aligns with literature emphasizing that achieving and maintaining the best possible vision should remain the primary goal in managing nAMD. Therefore, adherence to treatment schedules and the prompt identification of disease recurrence through appropriate monitoring are critical to optimizing visual outcomes (29). Communicating to patients that maintaining regular injections can preserve not only their vision but also their independence and quality of life may help enhance adherence (30, 31).

Similar results were obtained by other authors, who also demonstrated that patients with nAMD experienced greater severity of metamorphopsia compared to those with dry AMD (32). The most commonly self-reported metamorphopsia symptoms included distorted lines of words in books, newspapers, or on computer screens, as well as distorted window frames, bookshelves, and human faces. Image distortions caused by metamorphopsia constitute a significant impairment, resulting in progressive loss of reading ability, which can only be partially compensated with optical aids that facilitate eccentric viewing.

Reduced vision-related quality of life affects patients with retinal diseases such as nAMD, diabetic macular edema, and retinal vein occlusion who require intravitreal injections. Advanced age, the presence of dyslipidemia or depression, and poorer visual acuity are associated with a significantly lower quality of life during intravitreal therapy, underscoring the need to manage systemic comorbidities and psychosocial factors (29). Despite its inconvenience, intravitreal therapy is positively evaluated by patients, particularly when they recognize that the treatment helps prevent vision loss (33).

The goal of treating nAMD is long-term stabilization or improvement of visual acuity achieved by limiting disease activity, as reflected by the normalization of anatomical parameters. Intravitreal anti-VEGF injection therapy enables this goal to be met, allowing visual acuity to be maintained over an extended period—at least 4–5 years—often with long treatment-free intervals lasting several years (34, 35). Sustained preservation of visual function is of paramount importance, as it directly contributes to the maintenance of patients’ independence and overall quality of life. By preventing the progressive visual decline associated with untreated nAMD, effective long-term therapy enables patients to carry out daily activities, maintain social engagement, and reduce the risk of loss of autonomy commonly associated with visual impairment.

From a healthcare system perspective, the results highlight the importance of structured treatment programs and regular monitoring in optimizing patient outcomes. Ensuring timely access to intravitreal therapy and minimizing treatment delays may have a direct impact on both clinical outcomes and patient-reported quality of life. These findings may be particularly relevant for healthcare policy and resource allocation, as they underscore the value of integrating patient-reported outcomes into routine clinical practice and program evaluation.

Importantly, this study adds to the growing body of evidence by highlighting the role of contrast sensitivity and subjective visual disturbances as independent contributors to VRQoL, which are less frequently addressed in routine clinical assessments focused primarily on visual acuity.

Together, these findings emphasize that effective management of neovascular AMD should extend beyond anatomical and visual outcomes to include patient-centered measures, such as vision-related quality of life.

### 4.1 Strength and limitations

A limitation of our study is the sample size, which is not representative of the entire population of patients diagnosed with nAMD and may limit the statistical power and generalizability of the findings. However, it should be noted that the sample sizes in the studies used for comparison with our own were similar. Therefore, this limitation does not invalidate our findings.

A further limitation is that no formal a priori sample size calculation was performed; therefore, the study should be interpreted as an observational analysis of consecutively included patients rather than as a prospectively powered hypothesis-testing study.

A formal a priori power calculation was not performed, as the study sample was determined by the number of patients enrolled in the therapeutic program during the study period. This reflects the real-world nature of the study but may limit the statistical power to detect smaller effects.

The observational longitudinal design of this study should be considered when interpreting the findings. The absence of a control group limits the ability to establish causal relationships between treatment and observed improvements and introduces the possibility of regression to the mean. Patients included in the study were enrolled at the time of treatment qualification, which may partially explain improvements independent of the intervention.

Additionally, the single-center design may limit the external validity of the findings, as clinical practices, patient characteristics, and healthcare system factors can vary across institutions. Although the study was conducted in a well-defined and internally consistent clinical environment, which supports the reliability of data collection, this homogeneity may also limit the generalizability of the results. At the same time, it may reduce site-specific confounding, allowing for clearer attribution of observed effects to the variables under investigation.

The observation period in this study was limited to the duration of the therapeutic cycle defined by the national drug program. This reflects real-world clinical practice within the healthcare system in which the study was conducted, where treatment is structured into defined stages. However, it also limits the ability to assess long-term outcomes, including potential stabilization, plateau, or decline in vision-related quality of life over time.

There is also a potential for selection bias, as participants were recruited into a reimbursed drug program, which may preferentially include patients meeting specific eligibility criteria and thus not fully represent the broader population of individuals with nAMD. The allocation of patients to each anti-VEGF agent was not randomized, which may have also introduced selection bias and limited the comparability between subgroups.

Additionally, potential confounding factors such as baseline disease severity, comorbidities, and individual variability in disease progression were not fully controlled for in the statistical models. Patients with more advanced disease or specific comorbidity profiles may have been preferentially treated with one agent over another, potentially biasing comparative effectiveness estimates. Similarly, variation in baseline disease severity could independently affect response to treatment, thereby confounding observed associations. Although regression analyses were performed, they were limited to selected visual function parameters and did not include all clinically relevant covariates.

Treatment heterogeneity represents an additional limitation of the study. Patients received different anti-VEGF agents (aflibercept or brolucizumab). Although both agents share a common mechanism of action, they differ in molecular structure and duration of effect. These differences could have influenced both anatomical and functional outcomes, potentially confounding the observed treatment effects. However, due to the unequal distribution of patients between treatment groups, subgroup analyses were not performed. The lack of stratification by treatment type further limits the ability to assess drug-specific effects. Therefore, the results should be interpreted as reflecting overall treatment effects rather than drug-specific outcomes.

Another limitation concerns the method of collecting VRQoL data. Although the described survey technique ensured anonymity, there remains a risk of inaccurate responses due to conscious or unconscious manipulation by respondents, potentially caused by cognitive biases such as response bias and courtesy bias. Participants may have been inclined to provide responses they perceived as more acceptable or aligned with expected norms, particularly in domains involving subjective evaluations or sensitive topics. This issue is particularly relevant for participants who required assistance in completing the questionnaires due to reading difficulties. To mitigate these risks, the interview was conducted by the same person. Nevertheless, the possibility of response bias cannot be fully excluded. However, this limitation does not invalidate the study results, as the compared studies employed the same method and technique, and individual assistance was provided to respondents with near vision difficulties during questionnaire completion.

Among the strengths of this study is the fact that all ophthalmologic diagnostic assessments (visual acuity, presence and severity of metamorphopsia, and contrast sensitivity) were conducted by the same person. This approach minimizes the risk of inconsistent results and enhances the study’s reliability. Another strong point is the nearly equal representation of both sexes in the study group, which reduces the risk of bias and contributes to the overall quality of the research.

Another strength of the study is the use of the NEI VFQ-25 questionnaire to assess VRQoL, as it was also employed in the majority of studies used for comparison. The NEI VFQ-25 is a standardized, condition-specific instrument designed for individuals with ophthalmic disorders. It focuses specifically on VRQoL and is more sensitive to health status changes resulting from ophthalmologic conditions than general-purpose quality-of-life questionnaires.

### 4.2 Recommendations for further research

Future research should examine long-term VRQoL outcomes in larger cohorts to better understand how lasting the benefits of anti-VEGF therapy are. As new long-acting drugs emerge, clinical trials ought to compare their effects on visual function, treatment burden, adherence, and VRQoL with those of current standard regimens. Further studies could also explore whether incorporating low-vision aids, vision rehabilitation, or caregiver support programs enhances functional outcomes and VRQoL when combined with anti-VEGF treatment. Importantly, such research should consider both patient and caregiver perspectives, since many individuals depend on family members for transportation and everyday assistance. Future studies should incorporate multivariable models including clinical covariates such as disease stage, comorbidity burden, and treatment type to better isolate the independent determinants of VRQoL.

## 5 Conclusion

Vision-related quality of life in patients with neovascular age-related macular degeneration was low at baseline and showed a modest improvement following initiation of intravitreal anti-VEGF therapy over the follow-up period. Across time points, better visual acuity, higher contrast sensitivity, and fewer visual disturbances on the Amsler grid were associated with higher vision-related quality of life scores.

These findings support an association between visual function and patient-reported quality of life, but do not establish causality. While intravitreal therapy may contribute to maintaining functional vision, conclusions regarding broader impacts on quality of life and patients’ well-being should be interpreted with caution. Further multicenter studies with longer follow-up and controlled designs are warranted.

Clinically, achieving and maintaining the best possible visual acuity should remain the primary goal in managing neovascular AMD. Healthcare professionals are encouraged to inform patients about proposed therapeutic procedures, including their anticipated benefits. These benefits should include not only physical but also psychological aspects. This study highlights the potential psychosocial benefits of intravitreal therapy in nAMD and may help support patients in initiating treatment or in maintaining adherence to continuing therapy.

## Data Availability

The raw data supporting the conclusions of this article will be made available by the authors, without undue reservation.

## References

[1] Wong W Su X Li X Cheung C Klein R Cheng C et al. Global prevalence of age-related macular degeneration and disease burden projection for 2020 and 2040: a systematic review and meta-analysis. *Lancet Glob Health.* (2014) 2:e106–16. 10.1016/S2214-109X(13)70145-1 25104651

[2] Colijn J Buitendijk G Prokofyeva E Alves D Cachulo M Khawaja A et al. EYE-RISK consortium; European eye epidemiology (E3) consortium. Prevalence of age-related macular degeneration in Europe: the past and the future. *Ophthalmology.* (2017) 12:1753–63. 10.1016/j.ophtha.2017.05.035 28712657 PMC5755466

[3] Fraser C Lueck C. Illusions, hallucinations, and visual snow. *Handb Clin Neurol.* (2021) 178:311–35. 10.1016/B978-0-12-821377-3.00014-3 33832684

[4] WHOQOL Group. The World health organization quality of life assessment (WHOQOL): position paper from the Word heath organization. *Soc Sci Med.* (1995) 41:1403–9. 10.1016/0277-9536(95)00112-k 8560308

[5] Guyatt G Feeny D Patrick D. Measuring health-related quality of life. *Ann Intern Med.* (1993) 118:622–9. 10.7326/0003-4819-118-8-199304150-00009 8452328

[6] Talks S Daien V Mitchell P Aslam T Barratt J Biberger A et al. The patient voice in neovascular age-related macular degeneration: findings from a qualitative study. *Ophthalmol Ther.* (2023) 1:561–75. 10.1007/s40123-022-00631-7 36525220 PMC9756919

[7] Coleman A Yu F Ensrud K Stone K Cauley J Pedula K et al. Impact of age-related macular degeneration on vision-specific quality of life: follow-up from the 10-year and 15-year visits of the study of Osteoporotic fractures. *Am J Ophthalmol.* (2010) 5:683–91. 10.1016/j.ajo.2010.05.030 20691423 PMC2967587

[8] Mitchell J Bradley C. Quality of life in age-related macular degeneration: a review of the literature. *Health Qual Life Outcomes.* (2006) 4:97. 10.1186/1477-7525-4-97 17184527 PMC1780057

[9] Brown M Brown G Sharma S Stein J Roth Z Campanella J et al. The burden of age-related macular degeneration: a value-based analysis. *Curr Opin Ophthalmol.* (2006) 3:257–66. 10.1097/01.icu.0000193079.55240.18 16794438

[10] Flaxel C Adelman R Bailey S Fawzi A Lim J Vemulakonda G et al. Age-related macular degeneration preferred practice pattern^®^. *Ophthalmology.* (2020) 1:1–65. 10.1016/j.ophtha.2019.09.024 31757502

[11] Solomon S Lindsley K Vedula S Krzystolik M Hawkins B. Anti-vascular endothelial growth factor for neovascular age-related macular degeneration. *Cochrane Database Syst Rev.* (2019) 3:CD005139. 10.1002/14651858.CD005139.pub4 30834517 PMC6419319

[12] Finger R Daien V Eldem B Talks J Korobelnik J Mitchell P et al. Anti-vascular endothelial growth factor in neovascular age-related macular degeneration: a systematic review of the impact of anti-VEGF on patient outcomes and healthcare systems. *BMC Ophthalmol.* (2020) 1:294. 10.1186/s12886-020-01554-2 32680477 PMC7368708

[13] Gale R Finger R Eldem B Aslam T Barratt J Daien V et al. The management of neovascular age-related macular degeneration: a systematic literature review of patient-reported outcomes, patient mental health and caregiver burden. *Acta Ophthalmol.* (2023) 1:e26–42. 10.1111/aos.15201 35790079 PMC10084380

[14] Mangione C Lee P Gutierrez P Spritzer K Berry S Hays R. National eye institute visual function questionnaire field test investigators. Development of the 25-item national eye institute visual function questionnaire. *Arch Ophthalmol.* (2001) 7:1050–8. 10.1001/archopht.119.7.1050 11448327

[15] Roque A da Silva Borges GF Abe RY de Souza OF Machado MC Ferreira T et al. The effects of age-related macular degeneration on quality of life in a Brazilian population. *Int J Retina Vitreous.* (2021) 1:20. 10.1186/s40942-021-00290-z 33726848 PMC7962216

[16] Cruess A Zlateva G Xu X Rochon S. Burden of illness of neovascular age-related macular degeneration in Canada. *Can J Ophthalmol.* (2007) 6:836–43. 10.3129/i07-153 18026200

[17] Pauleikhoff D Scheider A Wiedmann P Gelisken F Scholl H Roider I et al. Neovascular age-related macular degeneration in Germany: encroachment on the quality of life and the financial implications. *Ophthalmologe.* (2009) 3:242–51. 10.1007/s00347-008-1797-9 18709375

[18] Schippert A Jelin E Moe M Heiberg T Grov E. The impact of age-related macular degeneration on quality of life and its association with demographic data: results from the NEI VFQ-25 questionnaire in a Norwegian population. *Gerontol Geriatr Med.* (2018) 4:2333721418801601. 10.1177/2333721418801601 30263908 PMC6149028

[19] Sørensen M Andersen S Henningsen G Larsen C Sørensen T. Danish version of the visual function questionnaire-25 and its use in age-related macular degeneration. *Dan Med Bull.* (2011) 6:A4290. 21651879

[20] Šiaudvytytė L Mitkutė D Balčiūnienė J. Quality of life in patients with age-related macular degeneration. *Medicina.* (2012) 2:109–11. 10.3390/medicina48020015 22491386

[21] Ismayilova I Turdaliyeva B Aldasheva N Veselovskaya N. Assessing the quality of life in age-related macular degeneration patients: a cross-sectional study in Kazakhstan. *Acta Biomed.* (2022) 6:e2022299. 10.23750/abm.v93i6.13580 36533748 PMC9828926

[22] Yuzawa M Fujita K Tanaka E Wang E. Assessing quality of life in the treatment of patients with age-related macular degeneration: clinical research findings and recommendations for clinical practice. *Clin Ophthalmol.* (2013) 7:1325–32. 10.2147/OPTH.S45248 23836961 PMC3702546

[23] Lee J Lin K Hou C Li P See L. Chinese version of the vision-related quality of life (NEI-VFQ-25) among patients with various ocular disorders: a pilot study. *Medicina.* (2022) 5:602. 10.3390/medicina58050602 35630019 PMC9147604

[24] Jager R Mieler W Miller J. Age-related macular degeneration. *N Engl J Med.* (2008) 24:2606–17. 10.1056/NEJMra0801537 18550876

[25] Inoue M Arakawa A Yamane S Kadonosono K. Intravitreal injection of ranibizumab using a pro re nata regimen for age-related macular degeneration and vision-related quality of life. *Clin Ophthalmol.* (2014) 8:1711–6. 10.2147/OPTH.S68293 25228787 PMC4160327

[26] Gomi F Migita H Sakaguchi T Okada H Sugawara T Hikichi Y et al. Vision-related quality of life in Japanese patients with wet age-related macular degeneration treated with intravitreal aflibercept in a real-world setting. *Jpn J Ophthalmol.* (2019) 6:437–47. 10.1007/s10384-019-00687-2 31673841

[27] Schroeder M Rung L Lövestam-Adrian M. No improvement in injection frequency or in visual outcome over time in two cohorts of patients from the same Swedish county treated for wet age-related macular degeneration. *Clin Ophthalmol.* (2017) 11:1105–11. 10.2147/OPTH.S130182 28652696 PMC5472435

[28] Rung L Lövestam-Adrian M. Three-year follow-up of visual outcome and quality of life in patients with age-related macular degeneration. *Clin Ophthalmol.* (2013) 7:395–401. 10.2147/OPTH.S41585 23467557 PMC3589196

[29] Flindris K Chatzipetrou C Papafotiou E Kaliardas A Koumpoulis I Melissourgos I. Quality of life and treatment satisfaction in patients receiving intravitreal injection therapy for neovascular age-related macular degeneration, diabetic macular edema, and retinal vein occlusion: a cross-sectional study. *Cureus.* (2025) 8:e91004. 10.7759/cureus.91004 41018360 PMC12461233

[30] Kolačko Š Predović J Tomić A Oršulić V. Life quality in patients with impaired visual acuity undergoing intravitreal medication applications. *Int J Environ Res Public Health.* (2023) 4:2879. 10.3390/ijerph20042879 36833575 PMC9956309

[31] Matamoros E Maurel F Léon N Solomiac A Bardoulat I Joubert M et al. Quality of life in patients suffering from active exudative age-related macular degeneration: the EQUADE study. *Ophthalmologica.* (2015) 3:151–9. 10.1159/000433448 26337381

[32] Xu K Gupta V Bae S Sharma S. Metamorphopsia and vision-related quality of life among patients with age-related macular degeneration. *Can J Ophthalmol.* (2018) 2:168–72. 10.1016/j.jcjo.2017.08.006 29631830

[33] Bian W Wan J Tan M Su J Yuan Y Wang Z et al. Predictors of health-related quality of life in Chinese patients receiving treatment for neovascular age-related macular degeneration: a prospective longitudinal study. *BMC Ophthalmol.* (2020) 20:291. 10.1186/s12886-020-01561-3 32677913 PMC7364534

[34] Gerding H. Langzeitergebnisse der intravitrealen anti-VEGF-Injektionstherapie bei feuchter altersabhängiger Makuladegeneration: eine Meta-Analyse [Long-term results of intravitreal anti-VEGF injections in wet AMD: a meta-analysis]. *Klin Monbl Augenheilkd.* (2016) 4:471–4. German. 10.1055/s-0041-111835 27116511

[35] Korva-Gurung I Kubin A Ohtonen P Hautala N. Visual outcomes of anti-VEGF treatment on neovascular age-related macular degeneration: a real-world population-based cohort study. *Pharmaceuticals.* (2023) 7:927. 10.3390/ph16070927 37513839 PMC10384898

